# A Case of a Constricted Vessel: The Impact of Acute Myeloid Leukemia on the Superior Vena Cava

**DOI:** 10.7759/cureus.49616

**Published:** 2023-11-28

**Authors:** Paavan Desai, Dhruvish Mistry, Jhanvi Kothari, Ashima Gupta, Keerthana Panchagnula, Gurinder Singh, Aakash Baskar, Yashash Pathak

**Affiliations:** 1 Internal Medicine, Gujarat Adani Institute of Medical Sciences, Bhuj, IND; 2 Internal Medicine, Gujarat Medical Education & Research Society (GMERS) Medical College and Hospital, Gandhinagar, IND; 3 Internal Medicine, Dr. Panjabrao Alias Bhausaheb Deshmukh Memorial Medical College, Amravati, IND; 4 Internal Medicine, Kasturba Medical College, Manipal, Bengaluru, IND; 5 Internal Medicine, Universidad Latina de Panama, Panama, PAN; 6 Internal Medicine, K.A.P. Viswanatham Government Medical College, Tiruchirappalli, IND; 7 Internal Medicine, Baylor St. Luke’s Medical Center, Houston, USA

**Keywords:** acute myeloid leukemia (aml), daunorubicin, cytarabine, atypical hematolymphoid malignancy, cervical mediastinoscopy, superior vena cava (svc) syndrome

## Abstract

Acute myeloid leukemia (AML) is the most prevalent form of leukemia in adults, with rising global incidence rates. AML usually presents with non-specific clinical features such as pallor, fever, and bleeding. This case report discusses a unique presentation of AML, where a 25-year-old female with a history of hypertension presented with unilateral facial swelling, chest pain, and shortness of breath. Radiologic investigations revealed a mediastinal mass encasing the superior vena cava (SVC), confirming the suspicion of SVC syndrome. Upon testing with a biopsy, the mass was found to be composed of immature myeloid cells confirming the diagnosis of myeloid sarcoma-associated AML. The patient’s treatment involved a combination of surgical debridement, induction chemotherapy, supportive care, and management of complications. This case highlights that despite its common occurrence, AML may present with atypical clinical manifestations such as SVC syndrome, posing challenges in its diagnosis and timely management.

## Introduction

Acute myeloid leukemia (AML) is the most common type of leukemia in adults with a global incidence of 119.57 × 10^3^ cases which has increased by 87.3% from 1990 to 2017. The ratio of males to females affected by the disease is 1.38:1 [1,2].

Common clinical features of AML include fatigue, pallor, fever, bleeding, and malaise. Less frequent symptoms include weight loss, weakness, dyspnea, palpitations, and bone pains. A physical examination usually shows hepatosplenomegaly, lymphadenopathy, and gum hyperplasia [3].

Superior vena cava (SVC) syndrome comprises a constellation of clinical signs and symptoms caused by obstruction of blood flow through the SVC, commonly resulting from thrombus formation or tumor infiltration of the vessel wall. This syndrome is most commonly seen secondary to malignancy, although recently there has been a rise in benign etiologies [4,5]. Regardless of etiology, face and/or neck swelling is the most frequent presenting sign, seen in almost all cases of SVC syndrome. Patients can also present with upper extremity swelling, dyspnea, cough, and headache [6].

This case report describes a patient who presented with symptoms of unilateral facial swelling, shortness of breath, and chest pain. Upon further investigation, a CT scan of the chest revealed a mediastinal mass that was causing compression of the SVC, leading to the patient’s symptoms. Laboratory findings showed the presence of AML in the patient. This report discusses the diagnosis, treatment, and outcome of this rare presentation of AML. Understanding the presentation and management of rare cases like this can improve clinical decision-making and patient outcomes.

## Case presentation

A 25-year-old female with a history of hypertension presented to the emergency department (ED) with worsening facial swelling, chest pain, and shortness of breath which started approximately two days ago. Notably, the patient had a family history of hypertension, with both the paternal grandmother and father having hypertension. At the time of presentation, the patient was normotensive and not on any hypertension medications. She first noticed bilateral arm pain in January for which she got an X-ray and blood tests done at an outside hospital (OSH). She developed chest pain a few days later and went to another ED, where she discovered that she had a mediastinal mass. Upon further workup with a CT scan, chest X-ray, and blood work, the mass was thought to be benign, and no further workup was recommended. Over the next few days, the patient experienced increasing chest pain and dizziness. She started having bluish discoloration of lips and breathing difficulty that was exacerbated with exertion. She reported having headaches and facial swelling every morning, along with right-sided numbness. Finally, as the facial swelling worsened, she presented to our ED for evaluation and was admitted to the hospital with suspicion of SVC syndrome. Normal blood counts and CT of the chest conducted at the OSH were accessed to analyze the mediastinal mass which showed a 6.6 x 6.2 x 7 cm anterior superior mediastinal mass.

**Figure 1:**
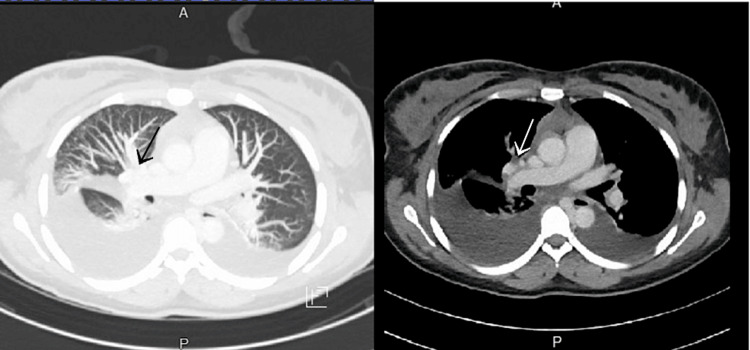
CT scan of the chest with and without contrast demonstrating a mass in the anterior superior mediastinal region which is encasing around the brachiocephalic veins and superior vena cava (SVC), accompanied by bilateral pleural effusion which is more pronounced on the right side.

Her complete blood count and flow cytometry on the first day of admission revealed 30% blast cells raising concerns for hematologic malignancy. Other notable laboratory findings on the first day were raised alkaline phosphatase (153 U/L), aspartate aminotransferase (56 U/L), blood urea nitrogen (5 mg/dL), chloride (108 mEq/L), and normal partial thromboplastin time (PTT) (28.2 seconds) and international normalized ratio (1.2). Flow cytometry was performed which revealed 30% circulating myeloblasts positive for CD34, CD13, CD33, myeloperoxidase (MPO), human leukocyte antigen - DR isotype (HLA-DR), and CD7. CT imaging of the chest showed a 6.6 x 6.2 x 7.0 cm anterior superior mediastinal mass encasing the bilateral brachiocephalic veins and SVC causing these central veins to collapse, confirming the diagnosis of SVC syndrome (Figure 1). The right brachiocephalic artery and right internal mammary artery were seen traversing the mediastinal mass. Multiple small nodules measuring up to 5 mm were also noted in the upper and middle lobe of the right lung, and bilateral pleural effusion was noted which was worse on the right side.

Given her blood reports and imaging results, hemato-oncology was consulted. AML, specifically acute promyelocytic leukemia (APL), was considered due to increased MPO and side scatter on flow cytometry, both of which were indicative of APL. However, the promyelocytic leukemia retinoic acid receptor alpha (PML RARA) fluorescent in situ hybridization and break-apart probe were both negative. These tests look for a specific genetic translocation (t 15;17) that results in the PML-RARA fusion gene, a hallmark of APL. The negative results suggested that this specific subtype of AML (APL) was unlikely. This indicated that the patient had a different subtype of AML, not APL.

The mass in the mediastinum was suspected to be a myeloid sarcoma. To confirm this, the patient underwent cervical mediastinoscopy and biopsy of the mediastinal mass which revealed a highly atypical hematolymphoid malignancy that was positive for CD45, CD43, and MPO but negative for CD34, CD117, CD3, and CD20, making it highly suggestive of myeloid sarcoma. Bone marrow examination showed 60% involvement with AML and cytogenetic abnormalities such as loss of chromosome X and 16, trisomy 13, isochromosome 17 with loss of 17p, gain of 17q, and six cells with trisomy 22.

As myeloid sarcoma is very chemosensitive, seven-day cytarabine and three-day idarubicin induction chemotherapy was started for myeloid sarcoma on the 22nd day. On the 13th day, the patient underwent debridement of the necrotic mass with the objective of eliminating the accumulated necrotic fluid.

The hospital course was complicated by (1) disseminated intravascular coagulation (DIC) that ensued as a consequence of the chemotherapy. This was managed by transfusing cryoprecipitate and fresh frozen plasma to the patient. On the 32nd day, an elevated PTT of 36.1 seconds was recorded. (2) Tumor lysis syndrome (TLS) was another complication that arose. The patient was administered allopurinol, received intravenous hydration, and underwent regular monitoring of electrolyte levels as part of the treatment protocol. (3) Pneumonia, secondary to neutropenic fever, was another complication. This was identified on a CT scan of the chest, which revealed ground-glass opacities in the right lung. The patient was administered a treatment regimen of vancomycin and meropenem for seven days. This treatment was initiated on the 24th day and concluded on the 30th day. Following this, the patient’s treatment was transitioned to prophylactic levaquin. Prophylactic fluconazole and acyclovir were also administered. (4) Bilateral pleural effusion, which was more pronounced on the right side. A CT scan of the chest revealed increased tree-in-bud opacities in the right upper lobe, prompting a thoracentesis. (5) Bilateral cephalic vein thrombosis was detected on an upper extremity Doppler ultrasound on the seventh day. However, there was no evidence of deep vein thrombosis.

The radiological evaluation indicated an improvement in the mediastinal mass. A repeat CT scan of the chest, performed on the 30th day, demonstrated a reduction in the mass dimensions to 5.1 x 5.5 cm. Throughout her hospitalization, the patient was given supportive care and her blood counts were monitored closely. The patient was discharged when she was hemodynamically stable, and her treatment plan included future consolidation therapy with high-dose cytarabine as an outpatient treatment. The focus was on achieving a good response to chemotherapy and considering the potential need for allogeneic hematopoietic stem cell transplantation (alloHSCT). The patient was provided with detailed information about alloHSCT, including further steps, anticipated toxicities of alloHSCT, the importance of finding a suitable donor, and the possibility of considering clinical trials pre- or post-transplant. The timeline and course of hospitalization for transplant were discussed, along with the risk of relapse even after transplantation. The patient was referred for HLA typing and donor search, and a follow-up to discuss transplantation was arranged once the donor search was complete.

## Discussion

AML is a type of blood cancer that starts in the bone marrow and can spread to other organs in the body such as lymph nodes (11.5%), spleen (7.3%), liver (5.3%), skin (4.5%), gingiva (4.4%), and central nervous system (1.1%) [7]. A very rare presentation of AML is the presence of the formation of a myeloid sarcoma which is a mass of immature myeloid or monocytic cells or precursors presenting in an extramedullary site [8]. The most common site for myeloid sarcoma reported in the literature is the mediastinum [9]. Among the etiologies of SVC syndrome, AML has a prevalence of around 1%, making it one of the most uncommon causes of this syndrome [10]. Optimal therapy for AML with myeloid sarcoma has not been validated yet due to the rarity of the condition. Usually, for the extramedullary location of AML in non-emergency cases, the primary treatment includes chemotherapy for sensitive malignancy, as done in this case. The existence of myeloid sarcoma accompanied by marrow involvement suggests the need for chemotherapy and the consideration of hematopoietic stem cell transplantation (HSCT). Radiotherapy is an option for individuals with solitary myeloid sarcoma, insufficient response to chemotherapy, recurrence following HSCT, and situations demanding prompt symptom relief due to compression of vital structures [11]. As literature is scarce on this presentation of the disease, documentation of management is important for future analysis for optimal management.

This is a rare case because of the patient’s presentation of AML as SVC syndrome from mediastinal mass compression. Moreover, the patient developed several complications during her hospital stay, including TLS, DIC, and necrotic fluid collection in the neck. The patient’s medical issues were managed with various interventions, including chemotherapy, surgery, and supportive care. Despite these challenges, the patient was eventually discharged upon hemodynamic stabilization and managed as an outpatient pending improvement in counts and repeat bone marrow biopsy. The patient was also referred for HLA typing and donor search for alloHSCT.

This case report highlights the complexity of managing a patient diagnosed with AML, SVC syndrome, TLS, DIC, neutropenic fever, and necrotic fluid collection. Furthermore, the case emphasizes the importance of considering myeloid sarcoma in the differential diagnosis of mediastinal masses, particularly in patients with AML. It also highlights the potential for myeloid sarcoma to cause SVC syndrome and underscores the need for prompt evaluation and management of this complication. Finally, the case underscores the importance of venous thromboembolism prophylaxis in high-risk patients, particularly those undergoing invasive procedures.

## Conclusions

This case report underscores the diagnostic complexity and therapeutic challenges presented by atypical manifestations of AML, emphasizing the importance of a comprehensive and multifaceted approach to patient management. The unique presentation of myeloid sarcoma causing SVC syndrome in a young female with AML highlights the need for vigilance and consideration of malignancy in patients with unusual clinical features. Despite the initial benign interpretation of the mediastinal mass, progressive symptoms warranted further investigation, leading to the correct diagnosis and appropriate management. The combination of surgical intervention, induction chemotherapy, and supportive care while navigating complications such as DIC and TLS facilitated a positive outcome in this case.

The patient’s journey from symptom onset through to discharge, with plans for high-dose chemotherapy and potential HSCT, illustrates the critical role of timely intervention and aggressive management of AML complications. This case also serves as a reminder of the potential for AML to manifest outside of the bone marrow and the necessity for a thorough evaluation of extramedullary masses. Documentation of such rare presentations of AML contributes to the broader clinical knowledge base, assisting in the refinement of diagnostic strategies and therapeutic protocols. As we continue to encounter and treat rare and challenging cases, they become a valuable source of learning and a testament to the evolving nature of oncological care.
